# Innate Immune Cells in Liver Inflammation

**DOI:** 10.1155/2012/949157

**Published:** 2012-08-09

**Authors:** Evaggelia Liaskou, Daisy V. Wilson, Ye H. Oo

**Affiliations:** ^1^Centre for Liver Research & NIHR BRU in Liver Disease, Institute of Biomedical Research, University of Birmingham, Birmingham B15 2TT, UK; ^2^Respiratory Medicine Department, University Hospitals Birmingham NHS Foundation Trust, Edgbaston, Birmingham B15 2TH, UK

## Abstract

Innate immune system is the first line of defence against invading pathogens that is critical for the overall survival of the host. Human liver is characterised by a dual blood supply, with 80% of blood entering through the portal vein carrying nutrients and bacterial endotoxin from the gastrointestinal tract. The liver is thus constantly exposed to antigenic loads. Therefore, pathogenic microorganism must be efficiently eliminated whilst harmless antigens derived from the gastrointestinal tract need to be tolerized in the liver. In order to achieve this, the liver innate immune system is equipped with multiple cellular components; monocytes, macrophages, granulocytes, natural killer cells, and dendritic cells which coordinate to exert tolerogenic environment at the same time detect, respond, and eliminate invading pathogens, infected or transformed self to mount immunity. This paper will discuss the innate immune cells that take part in human liver inflammation, and their roles in both resolution of inflammation and tissue repair.

## 1. Introduction

The immune system is made up of a coordinated network of cells, tissues and organs, which are able to attack non-self-exogenous pathogens and self-endogenous danger with a complex set of defence mechanisms. It responds to pathogens in two fundamental pathways: the primal strategy of “identifying and destroying” (innate immunity) or the specific detection and targeted killing process with regulation and memory (adaptive immunity) [1].

The innate immune system is the first line of defence against initial invading organisms and environmental challenges during the initial critical hours and days of life [2]. The overall survival of the host depends on its ability to recognise and induce the appropriate defence signals for the elimination of infectious microbes. Through anatomical barriers (skin and mucosal epithelia of the gastrointestinal, respiratory and reproductive tracts), soluble antimicrobial factors (acute phase proteins, complement and cytokines), and cellular components, the innate immune system provides protective barriers between the inside of the body and the outside world. 

Innate immune cells [monocytes, macrophages, mast cells, neutrophils and natural killer (NK) cells] are able to recognise pathogen associated molecular patterns (PAMPs) such as components of microorganisms [lipopolysaccharide (LPS), glycolipids, flagellin, lipoproteins, viral RNA and bacterial DNA] and endogenous ligands (such as heat shock proteins released by damaged or necrotic host cells) via their pattern-recognition receptors (PRRs), which include receptors for bacterial carbohydrates and toll-like receptors (TLRs). The TLRs and corresponding ligands, their impact on innate immune system are described in Table 1. Engagement of PAMPs with PRRs results in targeted and specific destruction of the activating organism, infected or tumour cells, by releasing cytotoxic agents or phagocytosis [3].

## 2. Liver as an Immunological Organ

Adult human liver is the largest internal organ in the body, weighing 1.2–1.5 kg. It has a dual blood supply with oxygenated blood entering through the hepatic artery (20%) and blood rich in nutrients and bacterial endotoxin entering the liver through the portal vein (80%). The arterial and portal-venous blood percolates through a network of liver sinusoids generating a mixed arterial-venous perfusion collected in the central vein and exit via three hepatic veins and drain back into the inferior vena cava [4, 5]. The liver is constantly exposed to antigenic loads of harmless dietary and commensal products from the gastrointestinal tract via portal vein and blood-borne antigens via hepatic artery. Thus, it is prerequisite for the liver immune system to be appropriately equipped in order to protect itself from pathogens and metastatic cells, whilst tolerating harmless self and foreign antigens. The liver innate cells (resident macrophages, named, Kupffer cells, dendritic cells, NK and NKT cells) and antimicrobial components (inflammatory cytokines, chemokines, acute phase proteins, complement) coordinate to achieve this critical task and eliminate invading pathogens and infected or transformed self [5].

In this paper, we will describe the innate immune cells phenotype, function in the context of human liver inflammation. 

## 3. Innate Immunity in Liver Inflammation

### 3.1. Acute Phase Proteins (APPs) and Complement System

#### 3.1.1. Acute Inflammation and Acute-Phase Proteins

During local liver injury or infection, resident Kupffer cells and monocyte/macrophages initiate an immune response. Upon phagocytosis of the pathogenic material, phagocytes release a variety of chemical messengers such as tumour necrosis factor alpha (TNF*α*), interleukin (IL)-1, and IL-6 that initiate the acute-phase response and inflammation. Such acute inflammation is characterised by the rise in concentration of numerous plasma proteins, collectively termed acute-phase proteins (APPs) [6]. APPs are a heterogeneous group of plasma proteins, which are exclusively synthesised in the liver and include pentraxins (C-reactive protein (CRP), serum amyloid P (SAP), and the long pentraxin 3 (PTX)), serum amyloid A (SAA), serum mannose-binding lectin, orosomucoid, inhibitors of proteases (*α*1-antitrypsin, *α*1-antichymotrypsin, *α*1-ACH, *α*2-macroglobulin), coagulation factors (fibrinogen, prothrombin, fVIII, plasminogen), transport proteins (haptoglobin, hemopexin, ferritin), and complement components [7]. The characteristic of these APPs is that their concentration can be increased (positive APPs) or decreased (negative APPs) by at least 50% in inflammatory disorders [8, 9].

APPs are critical components of the innate immune response restoring homeostasis after infection or inflammation. The important tasks they serve include haemostatic functions (e.g., fibrinogen), microbicidal and phagocytic functions (e.g., CRP and complement components), antithrombotic (e.g., *α*1-acid glycoprotein), and antiproteolytic properties which are required for maintaining protease activity at sites of inflammation (e.g., *α*2-macroglobulin, *α*1- antitrypsin and *α*1-antichymotrypsin) [10].

One of the major acute-phase proteins in humans is C-reactive protein. CRP belongs to the pentraxin superfamily of acute phase reactants that has originally been named for its ability to react with the C-polysaccharide of *Streptococcus pneumonia* [8, 11]. CRP production increases rapidly up to 1000-fold within 24–48 hours in response to infection, trauma, and tissue infection, and its concentration reduces the same rapidly after resolution of inflammation. Hence, the measurement of CRP is widely used to monitor various inflammatory conditions [8, 12]. CRP is produced mainly by hepatocytes, but it can also be produced by Kupffer cells, monocytes, and subsets of lymphocytes [11]. CRP binds to phosphocholine and phospholipid constituents of foreign pathogens and damaged cells and to chromatin in nuclear DNA-histone complexes, thus acts as an opsonin for various pathogens and activator of the complement system by binding to Fc receptors. Interaction of CRP with Fc receptors induces the production of proinflammatory cytokines that further enhance the inflammatory response. One characteristic of CRP is that it does not recognise specifically distinct antigenic epitopes, but recognises altered self and foreign molecules based on pattern recognition, thus provides early defence through production of proinflammatory signals and activation of the humoral and adaptive immune system [13]. *In vivo *studies in transgenic mice overexpressing CRP have confirmed its anti-inflammatory effects. Increased CRP could prevent the adhesion of neutrophils to endothelial cells by decreasing the surface expression of L-selectin, inhibiting the generation of superoxide by neutrophils and stimulating the synthesis of IL-1r*α* by mononuclear cells [8].

#### 3.1.2. Complement System

The complement system is a biochemical cascade of more than 35 proteins that plays an important role in innate immune defence against various pathogens through cytolysis, chemotaxis (e.g., C5a), opsonization (e.g., C3b), and activation of mast cells [14]. The complement system is activated through three different pathways: the classical, alternative, and mannose-binding lectin pathway. Its activation is initiated by the binding of one or more molecules of the above pathways on the surface of the target cells. The classical pathway destroys antibody-coated targets, apoptotic cells, Gram-negative bacteria, and some viruses. The alternative pathway destroys a variety of infectious agents including bacteria, viruses, and fungi in addition to playing a role in the immune surveillance of tumours, and the mannose-binding lectin pathway destroys mannose-bearing pathogens [15, 16]. All three complement activation pathways lead to the formation of C3 convertase, which in turn leads to the formation of membrane attack complex (MAC), a cytotoxic end-product of complement system made up of C5b, C6, C7, C8, and polymeric C9, that form a macromolecular pore capable of inserting itself into cell membranes and lysing heterologous cells, including bacteria and viruses, resulting in their death [16]. There is growing evidence suggesting that complement proteins not only serve as mediators of innate immune defence against foreign pathogens but can also modulate diverse developmental processes, such as cell survival, growth, and differentiation in various tissues [17]. The anaphylatoxins C3a and C5a, complement effector molecules released after complement activation, have been reported to be involved in the priming phase of liver regeneration, contributing to both the regulation of liver cell proliferation and hepatoprotection [17–19]. In complement deficient mice, lack of complement signalling results in impaired liver regeneration [19]. 

Depletion of serum complement before ischemia resulted in a significant attenuation of the KC-induced oxidant stress (enhanced oxidation of plasma glutathione) and also prevented the accumulation of PMNs in the liver during the reperfusion period suggesting that complement is involved in the induction of a KC-induced oxidant stress, the priming of KC and PMNs for enhanced reactive oxygen generation, and the continuous accumulation of PMNs in the liver during reperfusion [20]. Moreover, complement activation products can augment adhesion of leukocytes to endothelium, since C5b-9 and C5a can induce rapid translocation of P-selectin from Weibel-Palade bodies to the endothelial surface. The complement receptors CR3 and CR4 (CD18/CD11c) are members of the *β*-integrin family, which promote interactions between leukocytes and vascular endothelium [17].

### 3.2. Neutrophils

Neutrophils are polymorphonuclear cells that belong to the granulocyte family of leukocytes. They are the most abundant cells of the innate immune system and are indispensable for their defence against invading infectious pathogens. Neutrophils are generated in the bone marrow, where they remain for further 4–6 days, thus spending there the majority of their life [21, 22]. Their production is extensive in steady state with 1-2 × 10^11^ cells being generated per day in normal human adult [23]. In systemic circulation neutrophils form the majority of circulating leukocytes, but they only consist <2% of total neutrophils. They have a very short half-life (~6–8 hours in humans and ~11 hours in mice) and are generally functionally quiescent [24]. During episodes of infection, their number can be increased by up to 10-fold. In steady-state conditions, circulating neutrophils can home either to the spleen, liver, or return to the bone marrow to be destroyed [25]. Alternatively, in the event of a pathogenic invasion, neutrophils from peripheral blood are rapidly recruited into peripheral tissues to fulfill their primary role to eliminate microbial organisms. 

#### 3.2.1. Neutrophil Recruitment in Human Liver

A unique feature of the liver is that it has several anatomical compartments for leukocyte recruitment, including the endothelial cells lining hepatic sinusoids, and the endothelial cells lining the portal and terminal hepatic veins [4, 26]. Leukocytes are able to adhere and migrate across such different regions of the hepatic microvasculature, but the majority of these cells seem to enter the parenchyma via the hepatic sinusoids. The endothelial cells lining the hepatic sinusoids have distinct characteristics as they lack underlying basement membrane and tight junctions but have fenestra [27]. They display differences in adhesion molecule expression compared with other endothelial cells of central and hepatic veins. Adhesion molecules such as E- and P-selectin, which are expressed on endothelial surfaces of hepatic arteries, portal and central veins are absent in sinusoidal endothelial cells [28, 29].

Leukocyte recruitment (Figure 3) is a highly regulated process dependent on sequential interactions with endothelial adhesion molecules and chemokines. The initial interactions between endothelium and leukocytes induce tethering and rolling of the leukocyte on the endothelial surface via transient bonds between selectins and their glycoprotein ligands. This initial contact allows leukocytes to sample the endothelial microenvironment for chemokines, which can be secreted by the activated endothelium and immune cells and are immobilized by glycosaminoglycans on the endothelial cell surface. The binding of chemokines to chemokine receptors on leukocytes leads to rapid G-protein coupled signalling that triggers cytoskeletal rearrangement and activation of leukocyte integrins. The activated integrins are then able to bind to their ligands, members of the immunoglobulin superfamily expressed on the endothelial surface, hence firmly arresting the leukocyte on the endothelium. In the final step, leukocytes pass through the endothelial monolayer in a process named transendothelial migration or diapedesis, following directional cues to the site of infection or tissue injury [30, 31].

In the case of neutrophils, the initial step includes the slowing of this leukocyte within the venule. The cell is loosely tethered to the vessel wall and rolls along the endothelial surface at less than 50 *μ*m/sec velocity. Neutrophil rolling along the endothelium is mediated by the three members of the selectin family (E-, P-, and L-selectin) and their ligands. After rolling, neutrophils are firmly arrested on the endothelium via CD18 integrin/intercellular adhesion molecules (ICAMs) interactions. The adherent neutrophils migrate through the endothelial junctions into the region between the endothelium and its basement membrane. After stopping briefly at this location, neutrophils migrate into the surrounding tissue via *β*2-integrins (LFA-1, Mac-1) and ICAM-1 [32, 33]. This neutrophil recruitment cascade occurs in mesentery, brain, and skin *in vivo *and *in vitro.* However, some of the adhesion mechanisms in sinusoids may not be the same as in postcapillary venules.

However, the recruitment of neutrophils in the liver displays a different pattern [34]. Neutrophil recruitment and accumulation in the hepatic sinusoids is independent of selectins and *β*2-integrins, which are though required for their recruitment to the postsinusoidal venules [35–37]. It has been suggested that accumulation of neutrophils into the sinusoids is mediated by mechanical trapping of these cells in the narrow sinusoidal vessels due to changes of the activated neutrophils themselves, sinusoidal endothelial cell swelling, and additional low stress in these capillaries [38]. McDonald et al. [39] have supported that CD44 and its hyaluronan ligand (HA), which is extensively expressed on the sinusoidal endothelial cells, are responsible for neutrophil recruitment in liver sinusoids, as proven by blocking antibodies directed against either CD44 and HA. Recent reports have also highlighted the CD44/HA interaction as the dominant mechanism for neutrophil adhesion in sinusoids during endotoxemia and ischemia reperfusion [39, 40]. Although the adhesion molecules are the important “tracks” for neutrophil movement, their driving forces however are the chemotactic factors that induce their migration from systemic circulation to the site of infection. Such factors are cytokines (TNF*α*, IL-1*α*, and IL-1*β*), activated complement proteins, and CXC chemokine IL-8 (CXCL8, specific neutrophil chemoattractant) [32, 41].

#### 3.2.2. Neutrophil-Mediated Innate Immune Defence

Mature neutrophils are professional phagocytic granulocytes with numerous antimicrobial molecules (>300 proteins) stored in their cytoplasmic granules. These granules are unspecific molecules with high cytotoxicity and potential tissue-damaging activity that can be also involved in many neutrophilic processes including adhesion, migration, and antibacterial activities [42]. Thus, neutrophils are considered highly dangerous cells, whose action needs to be tightly controlled [43, 44]. This characteristic explains why neutrophils are mainly absent in tissues and body cavities in steady-state conditions and are predominantly kept in reserve pools as quiescent cells in the blood and bone marrow. This also explains the reason that they are the first cells to be recruited to the site of infection upon acute inflammation [45]. 

**Figure 1:**
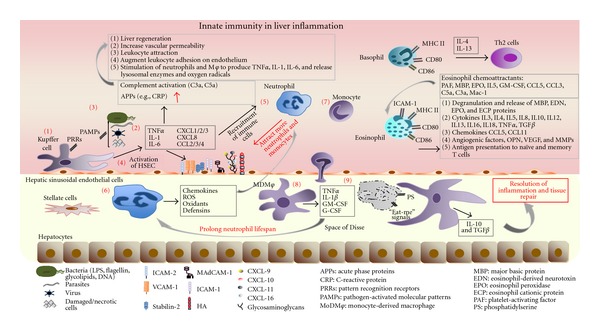
Innate immune cells in liver inflammation. During an infectious insult in the liver (1) resident macrophages, Kupffer cells, are the first immune cells to detect the presence of invading pathogens (bacteria, parasites, viruses, damaged, and/or necrotic cells) via PRRs/PAMPs. (2) Upon activation Kupffer cells release cytokines TNF*α*, IL-1, and IL-6 as well as chemokines CXCL 1–3, CXCL-8, CCL-2–4 that initiate (3) the acute-phase response and inflammation. Acute inflammation is characterized by the rise in plasma proteins, collectively named acute-phase proteins (APPs) that include C-reactive protein (CRP) and complement components. (4) Proinflammatory cytokines released from activated Kupffer cells can activate hepatic sinusoidal endothelial cells to upregulate adhesion molecules (ICAM1 and 2, VCAM-1, MAdCAM etc.) and in combination with the chemokines secreted from Kupffer cells can stimulate the recruitment of neutrophils and monocytes to the liver. (5) Neutrophils are the initial phagocytes to arrive at the site of microbial invasion, where (6) they change their phenotype, they become activated and release powerful and cytotoxic antimicrobial molecules such as reactive oxygen species (ROS), oxidants, defensins, as well as chemokines to attract more neutrophils and monocytes. (7) Following their recruitment to the tissue, monocytes undergo differentiation into (8) tissue macrophages (MDM*φ*), which release TNF*α*, IL-1*β*, G-CSF, and GM-CSF factors that can extend the lifespan of neutrophils thus sustaining their presence at the site of inflammation. (9) In order for inflammation to be resolved, the dangerous neutrophils at the inflammatory loci undergo apoptosis and terminate the inflammatory process quickly. Apoptotic neutrophils represent an important anti-inflammatory stimulus to other cells involved in the resolution of inflammation by producing “eat-me” signals recognised by the surrounding phagocytes. Phosphatidylserine (PS) residues on the apoptotic neutrophil membrane allow recognition by its receptor on macrophages, which not only initiates phagocytosis but also modifies the transcriptional profile of the M*φ*, increasing the production of IL-10 and TGF-b, cytokines associated with resolution of inflammatory response and tissue repair. Basophils are short-lived cells that express MHC II and CD80/CD86 costimulatory molecules, thus are able to present antigens to CD4+ T cells promoting their differentiation into Th2 cells via release of IL-4 and IL-13. Eosinophils recruited to the liver release proinflammatory mediators including granule-stored cationic proteins, cytokines, and chemokines. They also express MHC II, CD80/CD86, CD40, and ICAM-1; thus they are able to present antigens to T cells initiating or amplifying antigenic-specific immune responses.

During an infectious insult in the liver, resident macrophages and dendritic cells detect the presence of invading pathogens (via PRRs/PAMPs mechanisms) and will release chemokines CXCL8 (IL-8), CXCL1, 2, 3, CCL2, 3, 4 to attract neutrophils and monocytes at the site of infection (Figure 1) [44, 46, 47]. Neutrophils are the first phagocytes to arrive at the foci of microbial invasion, where they change their phenotype, become activated, and release cytotoxic antimicrobial molecules (reactive oxygen species (ROS), oxidants, defensins, lactoferrin and cathelicidins) [42, 48–51] as well as chemokines to attract primarily more neutrophils as well as monocytes, which extend the lifespan of the former from 6–12 hrs (at resting state) to 24–48 hrs at the inflammatory sites [45] by factors such as IL-1*β*, TNF*α*, G-CSF and GM-CSF [52]. 

In order for the infection to be effectively controlled and resolved, the neutrophils that are present at the infectious foci need to undergo apoptosis, a mechanism that renders them functionally quiescent [53]. Apoptotic neutrophil itself represents an important anti-inflammatory stimulus to other cells by producing “eat me” signals recognised by the surrounding phagocytes to resolve the infection. Scannell et al. [54] have identified the release of annexin 1 by apoptotic cells as a soluble signal that promotes neutrophil phagocytosis by macrophages. Moreover, the exposure of phosphatidylserine (PS) residues on the apoptotic neutrophil membrane allows recognition of PS with its receptors on macrophages, which not only initiates phagocytosis but also modifies the transcriptional profile of the macrophage, increasing the production of IL-10 and TGF*β*, two cytokines associated with resolving the inflammatory response and promoting tissue repair [43, 55].

#### 3.2.3. Neutrophil-Mediated Liver Tissue Injury

Protective immunity is always beneficial when it is well contained and properly regulated. Excessive neutrophil accumulation at the site of liver tissue injury may contribute to pathology through relevant proinflammatory and tissue-damaging effects from these cytotoxic phagocytes [56]. Liver injury mediated by neutrophils has been reported in a number of experimental animal models such as Concanavalin (Con)A-induced hepatitis [57, 58], ischemia-reperfusion injury [59, 60], alcoholic hepatitis [61, 62], endotoxemia [63], and sepsis [64]. Although the neutrophils that are accumulated in sinusoids are partially activated and primed, they cannot cause liver injury. Prerequisite for their cytotoxicity is their extravasation and adherence to parenchymal cells via ICAM-1/Mac-1 interaction [38, 65]. Adherence to parenchymal cells triggers the formation of reactive oxygen species and release of proteases through degranulation [38]. Neutrophils generate superoxide through NADPH oxidase, and the resulting hydrogen peroxide can either directly diffuse into hepatocytes or generate an intracellular oxidant stress. Neutrophil myeloperoxidase also generates hypochlorous acid, a major oxidant that also diffuses into target cells leading eventually to hepatocyte death [66, 67]. The proteases cathepsin G and elastase can also cause parenchymal cell necrosis, as protease inhibitors have been shown to attenuate neutrophil-induced liver injury [32, 68]. Neutrophils are detected in acute liver injury such as alcoholic hepatitis. Recent study from Lemmers and colleague suggested that IL-17 secreted from Th17, a new lineage of T helper cells act on fibroblast which in turn secreted IL-8 to attract neutrophils to site of alcoholic hepatitis suggesting the link between adaptive and innate immune system via cytokine IL-17 [69].

### 3.3. Monocytes, Macrophages and Kupffer Cells

#### 3.3.1. Monocytes: Origin, Heterogeneity, and Function

**Figure 2:**
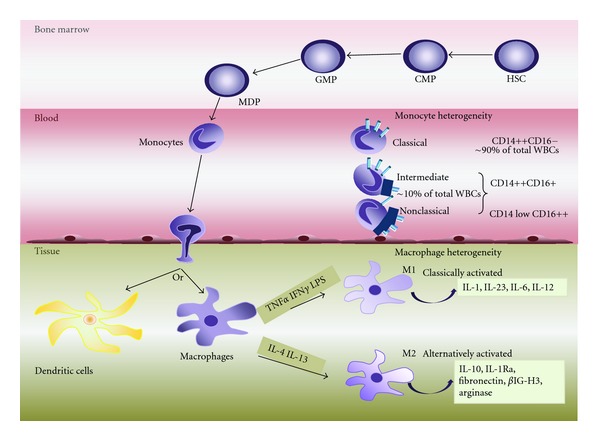
Monocyte and macrophage heterogeneity. Monocytes originate in the bone marrow where they develop from hematopoietic stem cells (HSCs) via several differentiation steps and intermediate progenitor stages that pass through the common myeloid progenitor (CMP), the granulocyte/macrophage progenitor (GMP), and the macrophage/DC progenitor (MDP) stages. The MDP gives rise to monocytes, which are released in blood circulation where they remain for 1–3 days. In peripheral blood, circulating monocytes represent ~5–10% of peripheral blood white blood cells (WBCs) and are a highly heterogenic population. Three main subtypes have been described based on the expression of CD14 and CD16 receptors: the classical CD14++CD16, intermediate CD14++CD16+, and nonclassical CD14 low CD16++ monocytes. In general, circulation monocytes are recruited to tissues where they can differentiate into dendritic cells or tissue macrophages (Kupffer cells in the liver; microglial cells in the brain, etc.), replenishing the existing populations. Additional heterogeneity also exists between the macrophages, with two major classes being identified: the classically activated (M1) and the alternatively activated (M2) macrophages. M1 macrophages are developed in response to TNF*α* and IFN*γ* as well as in response to microbial products such as LPS, and they produce in turn proinflammatory cytokines including IL-1, IL-23, IL-6, and IL-12. M2 macrophages can develop in response to IL-4 and IL-13 cytokines and play important roles in down-regulation of inflammation and tissue remodelling by releasing IL-10 and IL-1 receptor antagonist (IL-1Ra). They also produce high levels of arginase, fibronectin, and a matrix-associated protein, *β*IG-H3.

Monocytes originate from a common myeloid progenitor cell in the bone marrow that is shared with neutrophils. They are released in the bloodstream as nondifferentiated cells and circulate in the blood for 1–3 days [70]. Following recruitment to tissues, monocytes can differentiate into tissue macrophages (M*φ*s) or myeloid dendritic cells (DCs) [71–75], replenishing the existing populations and contributing to homeostasis maintenance, host defence, tissue remodeling, and repair [70, 76, 77] (Figure 2).

**Figure 3:**
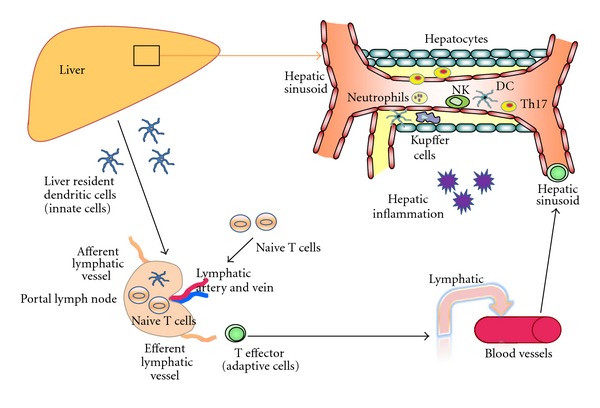
Innate immune cell (neutrophils, NK cells and monocytes) recruitment to hepatic inflammation. Human liver receives dual blood supply from both portal vein and hepatic artery. During the inital event of hepatic inflammation, innate immune cells such as neutrophils, monocytes and NK cells are recruited to the liver. Liver resident dendritic cells sample the foreign antigen and carry to local draining portal lymph nodes where antigens are presented to the adaptive naive T cells. Following the antigen presentation, different types of antigen-specific T effectors cells leave the nodes and drain back to systemic circulation. These T effector cells recruit via hepatic sinusoid towards the site of injury or inflammation. Th17 cells which secrete IL-17 attract neutrophils and also link innate and adaptive immunity.

Circulating monocytes constitute ~5–10% of peripheral blood leukocytes that show morphological heterogeneity [78]. The heterogeneity among human monocytes has been described since 1989 [79]. The differential expression of CD14 (part of the receptor for LPS) and CD16 (also known as Fc*γ*RIII) was initially traced in order to define two major subsets in peripheral blood: the so-called “classical” CD14++CD16 monocytes, typically representing up to 80% of the monocytes in a healthy individual, and the “nonclassical” CD16+ monocytes comprising the remaining fraction of monocytes (Figure 3) (paper in submission). It is now apparent that further heterogeneity exists and is that the nonclassical subset can be further divided into the intermediate CD14++CD16+ and the nonclassical CD14+CD16++ subpopulations. These subsets differ in many respects, including adhesion molecule and chemokine receptor expression [80, 81]. For mouse blood monocytes, a subdivision into three subsets similar to humans is also proposed that is classical, intermediate, and nonclassical. Specifically, in mouse the classical monocytes are Ly6Chi, CCR2hi, and CX_3_CR1low, whereas the nonclassical monocytes are Ly6Clow, CCR2low, and CX_3_CR1hi [81, 82].

Monocytes are members of the human mononuclear phagocyte system, which is important for the host nonspecific antimicrobial defence and tumour surveillance [82]. They are also a critical effector component of the innate immune system, equipped with chemokine receptors and adhesion molecules to recruit to site of infection. Monocytes secrete inflammatory cytokines, take up cells and toxic molecules, thus contributing to the immune defence against bacterial, protozoa, and fungal pathogens [83, 84]. Monocytes can kill bacteria by producing reactive nitrogen intermediates (RNIs), reactive oxygen intermediates (ROIs), and through the action of phagolysosomal enzymes [85]. 

#### 3.3.2. Monocyte Recruitment to Human Liver

Monocyte recruitment to the site of infection follows the general paradigm of leukocyte trafficking cascades, which involves rolling, adhesion, and transmigration. Monocytes are heterogeneous group and human monocyte subpopulations are defined on the basis of the expression of cell-surface markers. The classical CD14+ monocytes express high levels of CCR2 (the receptor for CCL2/MCP-1), low levels of CCR5 and low levels of CX_3_CR1. Conversely, CD16+ monocytes express high levels of CX_3_CR1 and CCR5 (receptors for CCL3/MIP1*α*). Therefore, both CX_3_CL1 and CCL3 are able to induce the transendothelial migration of CD16+ cells, whereas the recruitment of classical CD14+ cells depends on CCL2 [76, 86]. Additional studies in human peripheral blood monocyte subsets have shown that classical CD14++CD16− monocytes express CCR1, CCR2, CCR4, CCR5, CCR6, CXCR1, CXCR3, and CXCR5 chemokine receptors, whereas the nonclassical CD16+ monocytes show a limited chemokine receptor repertoire compared to CD14+ cells [87]. In mice, inflammatory monocytes express CD62L (L-selectin), LFA-1 (*α*L*β*2 integrin), Mac-1 (*α*M*β*2 integrin), PECAM-1 (CD31), and VLA-4 (*α*4*β*1). Therefore, initially, monocytes undergo CD62L selectin-dependent rolling along the vascular endothelium. Firm arrest is then mediated by integrins; interaction of *β*2 integrins with ICAM-2 causes firm arrest of monocytes in the absence of inflammation, whereas interaction of *β*2 integrins with their countereceptors ICAM-1 and ICAM-2 and of *α*4*β*1 with VCAM-1 mediates firm arrest and transmigration to inflamed tissues. Monocyte transendothelial migration across endothelium involves PECAM-1, CD99, CD226, and the junctional adhesion molecules (JAMs), which are present at tight junctions [88, 89]. After migration to the peripheral tissue, monocytes uses *α*4*β*1- and *α*6*β*1 integrins to interact with the extracellular matrix [82]. Previous study by Aspinall et al. from our group has reported that the recruitment of CD16+ monocyte subset to the inflamed human liver is mediated by VAP-1 and CX_3_CL1 [87].

#### 3.3.3. Monocyte-Derived Macrophages and Kupffer Cells in Human Liver

Inflammatory monocytes recruited at the site of inflammation can differentiate into macrophages. Tissue macrophages have a broad role in the maintenance of tissue homeostasis, through the clearance of senescent cells and the remodelling and repair of tissues after inflammation [90]. They are considered to be important immune effector cells that can clear approximately 2 × 10^11^ erythrocytes each day. Macrophages are also involved in the removal of cellular debris generated during tissue remodelling and rapidly and efficiently can clear the cells that have undergone apoptosis. The receptors involved in these homeostatic processes include scavenger receptors, phosphatidyl serine receptors, the thrombospondin receptor, integrins and complement receptors [91]. Moreover, necrosis that results from trauma or stress generates cellular debris that need to be cleared by macrophages. Phagocytosis of necrotic debris leads to dramatic changes in their physiology, including alterations in the expression of surface proteins and the production of cytokines and proinflammatory mediators. Macrophages are able to detect endogenous danger signals that are present in the necrotic cell debris through TLRs, intracellular PRRs, and IL-1R, most of which signal through the adaptor molecule MyD88. This function makes macrophages one of the primary sensors of danger in the host [91].

Additional heterogeneity also exists between the macrophages, with two major classes of macrophages being identified: the classically activated macrophages (M1) and the alternatively activated macrophages (M2) (Figure 2). M1 M*φ*s whose prototypical activating stimuli are IFN*γ* and LPS (which induces TNF production) generate proinflammatory cytokines, bactericidal mediators, and promote strong IL-12-mediated Th1 responses. In contrast M2 M*φ*s whose stimuli are IL-4 or IL-13 play an important role in the downregulation of inflammation supporting Th2-associated effector functions, tissue remodelling, elimination of tissue debris, and apoptotic bodies, as well as induction of angiogenesis [75, 92–94]. In general, macrophages are equipped with a broad range of pathogen-recognition receptors that make them efficient at phagocytosis and induce the production of inflammatory cytokines [84].

Kupffer cells (KCs), named after the pathologist C. von Kupffer are the liver resident macrophages which account for 80–90% of total fixed tissue macrophages in the body [95]. The origin of Kupffer cells has been speculated to involve two mechanisms: replenishment by local self-renewal and proliferation [96] and from circulating bone-marrow-derived monocytes. Kupffer cells are present throughout the liver residing within the lumen of liver sinusoids. Large KCs are mainly located in the periportal region where they are optimally located for response to systemic bacteria and bacterial products that are transported from the gut to the liver via the portal vein. Accordingly, periportal KCs have higher lysosomal enzyme activities together with greater phagocytic capacity than smaller KCs in midzonal and perivenous regions. Furthermore, large KCs produce higher levels of TNF*α*, PGE2, and IL-1 in contrast to the higher levels of nitric oxide formation by small KC [97, 98].

Kupffer cells are active phagocytes, which uptake intravascular debris, dead bacterial cells, and other blood-borne particles, and are able to secrete various inflammatory cytokines including IL-1, IL-6, TNF*α*, GM-CSF, and chemokines such as MIP-1*α* (macrophage inflammatory protein 1 alpha) and RANTES (regulated on activation, normal T-cell expressed and secreted). However, overproduction of such inflammatory mediators by Kupffer cells can lead to liver injury [99, 100]. Kupffer cells express several cell-surface receptor complexes involved in immune stimulation. These include complement receptors, Fc receptors, receptors for lectin-containing opsonins such as plasma mannose-binding lectin, adhesion receptors including those that bind ICAM-1, TLRs, and receptors for polysaccharides of microbial and host origin [101]. They also express high-affinity Fc*γ* receptors, which facilitate phagocytosis of IgG-coated particles, as well as receptors for IgA, galactose, and mannose receptors, and scavenger receptors which are capable of directly binding microbial surface components [101].

#### 3.3.4. Monocyte/Macrophage-Mediated Innate Immune Defence

Resident macrophages and dendritic cells are the first to detect the presence of invading pathogens by using invariant PRRs that recognise conserved PAMPs on extracellular and/or intracellular microbial components. Initially damaged cells spill cytoplasmic and nuclear components into the extracellular milieu, and these “alarm signals” activate tissue resident macrophages. CLEC4E is a transmembrane C type lectin, which has been reported to be involved in initiating the early inflammatory response after necrotic cell death [102]. The subsequent production of proinflammatory cytokines and chemokines including TNF, IL-6, CXCL1, CXCL2, CXCL3, CXCL8, CCL2, CCL3, and CCL4 can stimulate the recruitment of neutrophils and monocytes [103]. Granule proteins discharged from activated neutrophils anchor on endothelial proteoglycans and are recognised by monocytes that roll along the endothelium, thus promote their firm adhesion. Moreover, azurocidin, LL-37, and cathepsin G, proteases released from activated recruited neutrophils, activate formyl peptide receptors on classical inflammatory monocytes and promote their extravasation. Neutrophil granule proteins can promote *de novo* synthesis of monocyte-attracting chemokines by neighbouring endothelial cells and macrophages. In resolution of inflammation, apoptosis of neutrophils holds a central position as it brings to an end the sustained recruitment of neutrophils, while the phagocytic clearance of apoptotic neutrophils reprogrammes macrophages to an anti-inflammatory phenotype [104].

#### 3.3.5. Monocyte/Macrophage-Mediated Liver Tissue Injury

Monocytes/macrophages have an essential role in antimicrobial immune defence and are able to promote tissue healing and repair. However, they can also contribute to tissue destruction during some infections and inflammatory diseases [82]. The cytotoxicity of infiltrating macrophages or Kupffer cells has been reported in ischemia-reperfusion injury [105], endotoxemia [106], galactosamine hepatotoxicity [107], and corynebacterium parvum/endotoxin-induced liver injury [108]. It has been suggested that infiltrating macrophages and Kupffer cells mediate their cytotoxic effects through the production of reactive free radicals and specifically reactive oxygen species and proinflammatory cytokines including TNF*α*, IL-1*β* and IL-6. In addition, activated Kupffer cells can induce the infiltration of neutrophils. Again, proinflammatory cytokines released by Kupffer cells are thought to be important in the development of neutrophil-mediated tissue injury [59]. Previous study of Duffield et al. [109] demonstrated that deletion of macrophages either during injury or during repair and resolution has dramatically different effects on the overall fibrotic response. Specifically, in progressive inflammatory injury, macrophage depletion results in amelioration of fibrosis, whereas depletion during recovery results in a failure of resolution with persistence of cellular and matrix components of the fibrotic response. Hepatic macrophages have been implicated in APAP-induced liver hepatotoxicity (acetaminophen overdose), through the production of proinflammatory cytokines and mediators such as TNF*α*, IL-1*β*, and NO [110]. On the other hand, however, there are studies which described protective role of kupffer cells in acetaminophen-induced hepatic injury [111, 112]. The current concept suggests the role of macrophages predominantly in tissue repairs especially the newly recruited tissue macrophages [113].

### 3.4. Mast Cells

#### 3.4.1. Origin and Phenotype

 The mast cell is originally derived from the pluripotent haemopoetic stem cell. An immature version of the mast cell, an undifferentiated CD34^+^ and CD117^+^ progenitor cell, is released from the bone marrow into the blood stream [114, 115]. Mast cells are sessile and predominantly inhabit perivascular dermal and submucosal (respiratory/gastrointestinal/genitourinary tracts) connective tissue and lymph nodes [116]. They mature only once they have reached their tissue destination. The stem cell factor, c-kit, plays a critical role in the maturation process of the mast cell. Mast cells can be broadly divided into two categories, connective tissue mast cells, which are known as mast cell tryptase and chymase (MC^TC^) that release IL-4, and mucosal mast cells also known as mast cell tryptase (MC^T^) and produce IL-5 and IL-6 [117]. Once resident in the tissue, the mast cell has a life span of several months. They can proliferate, have a plasticity potential [115], and are mainly involved in Th2 immune response at the infected sites.

#### 3.4.2. Mast Cells in Innate Immune Response

Mast cells are among the first responders during infection that also provide immediate action by recruiting other immune cells to the scene of inflammation. Mast cells are large cells whose content is dominated by cytoplasmic granules. These cytoplasmic granules contain a variety of mediators including serotonin, histamine, cytokines, chemokines, and leukotriene. Histamine on its own composes 10% of the entire weight of the mast cell which illustrates the importance of the cytoplasmic granules to the function of the mast cell. 

Degranulation of mast cells and release of the mediators occur primarily via an IgE-mediated pathway but also via surface receptor binding sites including TLRs and *β*2 integrin. Mast cells have receptors, known as Fc*ε*RI, with high affinity for IgE on their surface. In fact the receptors have such high affinity for IgE that there is very little circulating IgE, as most is bound to mast cells already. The binding of IgE to Fc*ε*RI creates a sensitised mast cell ready to degranulate. The degranulation occurs when bi- or multi-valent antigen binds to the IgE causing cross-linking between the IgE. This leads to rapid exocytosis of the stored mediators, degranulation. This can also occur when substances such as neuropeptides and anaphylatoxins C3a and C5a bind to receptors on the mast cell surface. Toll-like receptor ligands can bind to toll-like receptors on the surface of mast cells and cause secretion, rather than degranulation of cytokines, chemokines, and lipid mediators [118]. 

Mast cells can amplify or suppress different areas of both innate and adaptive immunity depending on the concentration and type of the mediator released. The main mediators contained in the mast cell are histamine, heparin, cytokines, chemokines, and lipid mediators. Histamine and heparin are able to increase vascular permeability, cause smooth muscle contraction, and directly kill parasites. The major role of mast cells in innate immunity is to recruit neutrophils which can either enhance immune defence of host or can lead to immunopathology [118]. Lipid mediators are also involved in smooth muscle contraction, and can increase vascular permeability as well as neutrophil, eosinophil and platelet activation and mucus secretion. 

#### 3.4.3. Mast-Cell-Mediated Liver Tissue Injury

The number of mast cells within the liver is proportionately low in comparison to other tissues. The density of mast cells is calculated at between 1.2 and 3.9 cells per square millimetre of human liver. Hepatic mast cells are mostly situated within connective tissue adjacent to the hepatic artery, hepatic vein and bile ducts of the portal tract [119]. Recent studies have shown the role of intrahepatic mast cells in different chronic liver diseases [119]. Increased mast cell numbers have also been reported in liver fibrosis and hepatitis [120] and have been involved in acute hepatitis [121], primary biliary cirrhosis [122, 123], primary sclerosing cholangitis [123], hepatocellular carcinoma and cholangiocarcinoma [124, 125].

### 3.5. Basophils

Basophils are granulocytes that develop from hematopoietic stem cells in the bone marrow. They leave bone marrow after maturation, enter systemic circulation, and finally migrate to the inflammatory sites, where they play essential effector functions in response to parasite infection and allergic inflammation [126, 127].

#### 3.5.1. Origin and Phenotype

Basophils are short-lived cells (lifespan of 2-3 days) that account for less than 1% of circulating granulocytes in the blood [128]. However, their low baseline numbers can be expanded in response to growth factors such as IL-3, which has been reported to be important for basophil activation, population expansion, and survival [129]. Basophils express the high-affinity IgE receptor (Fc*ε*R1) present in a tetramer form (*αβγ*2) [130], and their activation can be induced in IgE-dependent (by IgE/Fec*ε*R1 interaction) and IgE-independent manner (by cytokines (IL-3, IL-6, IL-18, IL-33, TNF*α*, and GM-CSF), antibodies (IgG and IgD), allergens, parasite antigens, toll-like receptor (TLR) ligands and complement factors). Activation of basophils results in their degranulation and release of pro-formed (histamines) and newly synthesized lipid mediators, cytokines (IL-4, IL-13, IL-6, TNF*α*, and thymic stromal lymphopoietin (TSLP)) and chemokines, which are essential players in vascular reaction, exudation, leukocyte accumulation and wound healing [131, 132].

Basophils are mainly found in the blood and spleen and upon exposure to stimuli such as allergens or parasites they become activated. Activated basophils are then able to migrate to lymph nodes [133–135]. Basophils express a wide spectrum of chemoattractant receptors, such as cytokine receptors (e.g., IL-3R, IL-5R, GM-CSFR) [130, 136], chemokine receptors (CCR1, CCR2, CCR3, CXCR1, CXCR3 and CXCR4) [137–141], and receptors for more pleiotropic chemotactic factors such as receptors for complement components C3a and C5a, formyl-methionine-leucine-phebylalaning (fMLP), platelet-activating factor (PAF), leukotriene B4 (LTB4) [142–144]. Thus, basophils have the potential to respond to a wide variety of inflammatory stimuli, and some basophil populations migrate to draining lymph nodes, while others accumulate in inflamed tissues during an ongoing inflammatory response. 

#### 3.5.2. Basophil Recruitment and Function in Lymph Nodes and Tissues

Basophil recruitment from the peripheral circulation to the sites of infection occurs through the multistep process of leukocyte recruitment that has been described above. *In vitro* studies have shown that TNF*α* and IL-1 enhance basophil adhesion on endothelial cells, possibly through induction of basophil adhesion molecule expression. Moreover, it has been reported that IL-3 increases basophil adhesiveness to endothelial cells, possibly by increasing CD11b, an integrin that interacts with ICAM-1, fibrinogen and C3bi. CD11b and CD11c are also induced on the surface of basophils after activation [145]. 

Although for many years it has been well accepted that basophils are late-phase effector cells that migrate to the site of inflammation after the establishment of a Th2 cytokine response, recent studies have provided evidence that basophils can also play a central role in the induction and propagation of a Th2 cytokine-mediated immunity and inflammation [146, 147]. In the lymph nodes, basophils are able to directly interact with naive CD4+ T cells and induce their differentiation into Th2 cells. They express MHC class II and costimulatory molecules CD80 and CD86, thus basophils can present antigen via MHC class II and can provide IL-4 that promotes the differentiation of naïve T cells [147]. Basophils can also produce IL-13 upon stimulation with Ag/IgE complexes and can strongly release IL-4 and IL-13 in response to IL-3 and IL-18 or IL-33, further supporting their role in the development of Th2 cells [147]. Interestingly, independent groups have demonstrated that basophils are the predominant antigen-presenting cell (APC) in inducing Th2 responses against helminth parasites and allergens [133, 146, 148].

#### 3.5.3. Basophils in Liver Inflammation

Studies reporting the role of basophils in human liver inflammation are very limited. It has been described that infection with intestinal nematode *Nippostrongylus brasiliensis *induces robust Th2 immune responses and also enhances basophil generation in the bone marrow and subsequent accumulation in the peripheral tissues, including liver, lung, and spleen [149]. Further studies have also shown that basophils isolated from the spleen, liver or bone marrow are able to initiate Th2 cell development in the presence of antigens and DCs [150, 151].

### 3.6. Eosinophils 

#### 3.6.1. Origin, Phenotype, and Function

Eosinophils develop and mature in the bone marrow from multipotent hematopoietic progenitor cells of a myeloid lineage in IL-3, IL-5 and GM-CSF dependent manner. IL-5 has been described as the major lineage differentiation factor as well as the stimulus for eosinophils to leave the bone marrow and enter the circulation [152]. In the blood, mature eosinophils circulate for a short time (half-life of 8–18 hours), and then migrate out of the vessels into tissue. They consist approximately 1–3% of total circulating white blood cells, since a large pool remains in the bone marrow and the vast majority is located in the tissues, particularly at the mucosal surfaces of the gastrointestinal tract (lamina propria), mammary gland, respiratory and reproductive tracts [153–155].

#### 3.6.2. Eosinophil Recruitment to Tissue

Eosinophils express an array of cell surface molecules including immunoglobulin receptors for IgG (Fc*γ*RII/CD32) and IgA (FC*α*RI/CD89), complement receptors (CR1, CR3, and CD88), leukotriene receptors (CysLT1R and CysLT2R, LTB4 receptor), prostaglandin receptors (PGD2 type 2 receptor), platelet activating factor receptor (PAF), and toll-like receptors (particularly TLR7/8), cytokine receptors (IL-3R, IL-5R, GM-CSF that promote eosinophil development, as well as receptors for IL-1*α*, IL-2, IL-4, IFN*α*, and TNF*α*), chemokine receptors (CCR1 and CCR3) and adhesion molecules (VLA/*α*4*β*1, *α*4*β*7, Siglec-8) [130].

The migration of eosinophils from the blood into tissues involves selective adhesion pathways and chemoattractants. Chemoattractants for eosinophils include platelet-activating factor (PAF), complement component C5a [156], IL-16 [157], RANTES [158], MCP-3 [138] and eotaxin [159, 160]. Eosinophils can pass through post-capillary venules into tissues following chemoattractants in several steps of recruitment cascades of rolling, firm adhesion, and transendothelial migration. At the initial steps of tethering and rolling on endothelium, eosinophils make use of the receptors L-selectin, PSGL-1 and VLA-4 (*α*4*β*1), that interact with their counter receptors GlyCAM-1, CD34 and MAdCAM-1 (all L-selectin ligands), P-selectin and VCAM-1, respectively on the surface of endothelial cells [161, 162]. Following rolling, eosinophil integrins LFA-1 (CD11a/CD18), Mac-1 (CD11b/CD18), VLA-4 and *α*4*β*7 become activated and lead eosinophils to firmly arrest on ICAM-1, ICAM-2, VCAM-1 and MAdCAM-1, respectively on the endothelial surface [163]. In order to infiltrate into the tissue, eosinophils need to penetrate gaps between the endothelial cells. Utilizing Mac-1/ICAM-1 interactions and PECAM-1/PECAM-1 homotypical interactions between both cells at transendothelial junctions, eosinophils are able to translocate to the underlying basement membrane and through the extracellular matrix into the tissue [164, 165]. 

#### 3.6.3. Eosinophils in Innate Immune Defence

Activated human eosinophils are able to defend host against parasites, fungi and invading bacteria, by using functionally important receptors such as TLRs (TLR1, TLR4, TLR7, TLR9, and TLR10), responsible for recognition of conserved motifs in those pathogens [166]. Proteolytic enzymes released by various microbes and allergens, cross-linking of IgG or IgA Fc receptors, IL-3, IL-5, GM-CSF, CC chemokines and PAF mediators can potentially induce activation of eosinophils [130].

Eosinophils are characterised by their cytoplasmic granules that contain cationic proteins: major basic protein (MBP), eosinophil-derived neurotoxin (EDN), eosinophil cationic protein (ECP), and eosinophil peroxidase (EPO). These basic proteins play key roles in killing parasites, microorganisms, and tumour cells [156]. Degranulation of eosinophils can be induced by soluble stimuli, such as IL-5, GM-CSF, eosinophil-chemotactic cytokines CCL5 and CCL3, the lipid mediator PAF, the complement fragments C5a and C3a. The granule proteins, MBP and EPO acting in an autocrine manner, and the integrin Mac-1 which plays a role in eosinophil recruitment can also play a role in eosinophil degranulation [158, 167, 168].

At the sites of inflammation, recruited eosinophils release proinflammatory mediators including granule-stored cationic proteins, and newly synthesized eicosanoids, cytokines and chemokines including TGF*α*, TGF*β*, IL 3–5, IL-8, IL-10, IL-12, IL-13, IL-16, IL-18, TNF*α*, CCL-5 and CCL11 and profibrotic and angiogenic factors such as osteopontin, VEGF and MMPs [169–172]. They also promote Th2 responses. Eosinophils also possess the ability to internalise, process and present antigenic peptides within the context of surface-expressed major MHC class II. They express CD80, CD86, CD40 and ICAM-1 thus they are capable to provide costimulatory signals to T cells, present antigens to naïve and memory T cells and initiate/amplify antigen-specific immune responses. In healthy individuals, circulating eosinophils are devoid of MHC class II, but they are induced to express MHC II and costimulatory molecules upon exposure to appropriate cytokine stimuli and transmigration through endothelial cell monolayer [173–175].

IL-5, IL-3 and GM-CSF besides being growth and maturation factors for eosinophils, can also enhance several eosinophil functions. Th2 cytokines, IL-4 and IL-13 can also activate eosinophils. 

#### 3.6.4. Eosinophils in Liver Injury

Activated eosinophils have been suggested to play important roles in the pathogenesis of various liver diseases including primary biliary cirrhosis (PBC) [122, 176, 177]*; *primary sclerosing cholangitis (PSC) [178, 179] idiopathic hypereosinophilic syndrome [180, 181], drug-induced liver injury [182, 183], graft-versus-host disease [184], and hepatic allograft rejection [185–189]. Experimental studies have shown that activated eosinophils could play a critical role in the pathogenesis of liver diseases through the release of highly cytotoxic granule proteins such as MBP, ECP, TNF*α* followed by cell damage. The first experimental model to prove *in vivo* eosinophil-induced hepatotoxicity was established by Tsuda et al. in 2001 [190] by using IL-5 transgenic mice with a consequent blood hypereosinophilia. These mice after injection of LPS developed an extensive hepatic lobular necrosis, associated with a transmigration of eosinophils through vascular endothelium and degranulation of their cytotoxic granules in inflamed areas. These eosinophilic injuries were transient but liver specific. A recent study by Takahashi et al. [191] has also demonstrated an increased expression of galectin-9 and eosinophilic chemoattractant in the liver biopsy of patients with drug-induced liver injuries. Tarantino et al. [192] have reported an association between liver fibrosis and eosinophilia infiltrate (EI), which could be explained by the eosinophils' ability to release TNF-*α* and other cytokines capable of increasing an inflammatory cascade and therefore stimulating the fibrogenic stellate cells. 

### 3.7. Dendritic Cells (DCs)

#### 3.7.1. Phenotype and Function

Dendritic cells (DCs), first discovered [193] by Steinman, are professional antigen-presenting cells which control immunity and tolerance. They initiate and regulate immune responses depending on signals received from the invading microbes and their cellular environment. They are a heterogeneous population which can be divided into two major population; myeloid CD11c+ DCs (mDCs) expressing DC-SIGN and plasmacytoid CD123^+^ DCs (pDCs) which are also known as IFN producing cells [194, 195]. 

Myeloid DCs are HLA-DR^+^CD11c^+^ and express TLR 2, 3, 4, 5, 8. Myeloid DCs exist in three compartments; peripheral tissues, secondary lymphoid organs and in circulating blood. Peripheral tissue resident DCs consist of Langerhans cells (epidermis, gut) and dermal interstitial DCs [196]. Lymphoid organ resident DCs play a critical role in both induction of immunity to invading pathogens and maintenance of tolerance. They capture antigens and upon stimulation via pattern recognition receptors, they induce the proliferation of antigen-specific T cells. They are able to present antigens to CD4^+^ and CD8^+^ T cells as well as B cells. 

Plasmacytoid DCs are HLA-DR^+^CD123^+^, express TLR 7, 9, 10 and are present in blood, secondary lymphoid organs and peripheral tissues (skin and lungs) [197]. Their main function is to secrete IFN-*α* in response to viral infections and to prime T cells against viral antigens [198]. Plasmacytoid DCs are also described as tolerogenic DCs because they could induce regulatory T cells [199].

#### 3.7.2. Dendritic Cells in Innate Immunity

Both myeloid and plasmacytoid subsets are capable of initiating innate immune responses that lead to elimination of invading microbes. DCs express several receptors for recognising viruses including pattern recognition receptors (PRRs) such as the toll-like receptors (TLRs) and C-type lectins [200]. pDCs secrete large amount of type I IFN in response to viral encounter [201]. Activated mDCs produce cytokines such as interleukin-12, IL-15, and IL-18. IL-12 is crucial for mDCs to induce T helper 1 (Th1) cell responses, which subsequently promote the potent cytotoxic T lymphocyte (CTL) responses that are necessary for clearing microbe-infected cells [202]. 

DCs detect microbes in peripheral tissue sites and, following activation and microbe uptake, migrate to draining lymph nodes, where they promote NK cell activation. DCs also activate NKT cells to secrete IFN-*γ* and IL-4 [203]. DCs trigger different types of adaptive T-cells immune responses based on antigen and cytokine environment; they can promote IL-10 secreting regulatory T-cell development [204]; induce Th1 response [205] through upregulation of IL-12 secretion and Th2 responses [206] via secreting Th2 cytokines. 

#### 3.7.3. Dendritic Cells in Hepatic Inflammation

**Figure 4:**
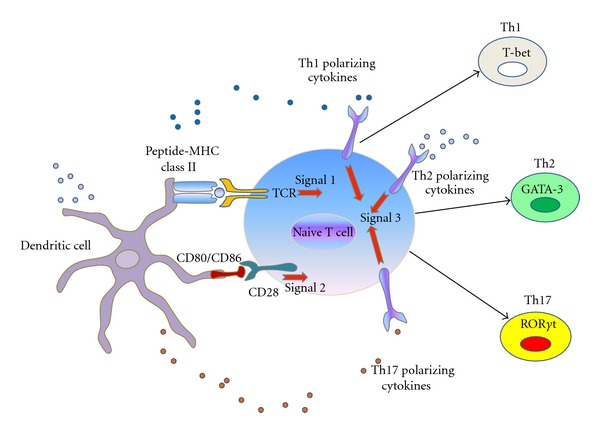
Linking innate and adaptive immune system. Dendritic cells from innate immune system present their antigen to naive T cells at local draining lymph nodes. T-cell receptor (TCR) ligation to MHC class II associated peptides processed from pathogens (signal 1) and binding of costimulatory molecule CD28 on lymphocyte to CD80 and CD86 expressed by dendritic cells (signal 2) leads to T-cell lineages differentiation. Signal 3 is the polarizing cytokines signals from the innate immune cells. Depending on type of antigen which is presented and nature of cytokines in the microenvironment, innate DC cells direct the development of Th1, Th2, Th17 lymphocytes lineages which plays crucial role in adaptive immune system.

Both plasmacytoid and myeloid DCs reside in the human liver. Hepatic DCs play important roles in the induction and regulation of immune responses (Figures 3 and 4). Human liver is constantly exposed to gut pathogens thus liver resident DCs remain in an immature state expressing low levels of MHC and costimulatory molecules CD40, CD80, and CD86. Intra-hepatic DCs tend to act as tolerogenic cells preferentially expressing IL-10 [207]. The constant exposure to bacterial LPS via portal blood down-regulates the expression of TLR4 on liver DCs thus limiting their response to danger signals and resulting in reduced or altered activation of the hepatic adaptive immune responses. DCs also have the capacity to expand functional CD4^+^CD25^+^ regulatory T cells [208, 209] and recent study has suggested that CCR9^+^ plasmacytoid DCs (pDCs) are capable of inducing regulatory T cells and inhibiting antigen-specific immune responses both *in vitro* and *in vivo* [210]. The role of DCs has been widely described not only in viral and autoimmune diseases but also in hepatocellular carcinoma and liver transplantation [211, 212].

### 3.8. Natural Killer (NK) Cells 

#### 3.8.1. Phenotype and Function

NK cells, first described as “pit cells” [213] are a crucial component of innate immune system. They are abundant in the liver where they provide a first line of defence against viral infections and tumour immunity [214, 215]. Hepatic NK cells in mice consist of 5–10% of lymphocyte population and they are defined by NK1.1^+^ (only for CD57BL/6 mice) CD3^−^ or DX5^+^ CD3^−^. In the human liver, NK cells consist approximately 20-30% of liver resident lymphocytes [216] and they are CD56^+^CD3^−^.

Human NK cells can be divided into two major populations; CD56^dim^ CD16^bright^ CD3^−^ and CD56^high^ CD16^dim^ CD3^−^. The former comprise approximately 90% of peripheral circulating NK cell population. They constitutively produce high numbers of cytolytic granules and are capable of spontaneously lysing target cells in the absence of prior sensitization. The latter consist the remaining 10% of circulating NK cells that are poorly cytotoxic and express high levels of C-type lectins and natural cytotoxicity receptors (NCRs) and low levels of killer cell immunoglobulin-like receptors (KIRs) [217]. These two NK cell subsets represent different stages of NK cell maturation, with the CD56^dim^ NK cells being the functionally and phenotypically mature cells [218]. A third population of NK cells consisting of CD56^−^ cells has been demonstrated during chronic viral infections [219]. They express a similar receptor profile to CD56^low^ NK cells but are poorly cytotoxic and do not secrete cytokines [220–222].

#### 3.8.2. NK Cell Recruitment in Liver

NK cells arrive very early to the site of inflammation and generally reside in the hepatic sinusoids. They express chemokine receptors CCR2 (which responds to chemokine CCL2), CCR5 (ligands are CCL5, CCL7, CCL8), CXCR3 (CXCL9-11), CX_3_CR1 (CX3CL1) and S1PR (SIP) thus responding to a variety of chemokines. Both CD56^dim^ and CD56^bright^ NK cell subsets migrate to inflamed sites with more CD56^dim^ being recruited to inflamed liver. Previous studies have suggested that Kupffer cell derived CCL2/MCP-1 recruits CCR2 expressing NK cells to the liver [223, 224]. During hepatic inflammation, activated liver sinusoidal endothelial cells express CXCL9-11 chemokines (CXCR3 ligands) [225] which subsequently recruit CXCR3 expressing NK cells to the liver. They also secrete chemokines CCL3/ MIP-1*α* and CCL4/MIP-1*β* which lead to subsequent T cells recruitment to the liver [226]. IFN-*γ* secreted from NK cells favours development of Th1 cells and upregulates CXCL9-11 chemokines (CXCR3 ligands) on human hepatic sinusoidal endothelium thus will recruit various inflammatory cells expressing CXCR3 chemokine receptors.

#### 3.8.3. NK Cells in Hepatic Inflammation

NK cells play a significant role in antiviral and antitumour activity, liver fibrosis, liver repair and may also be involved in hepatic tolerance. NK cells main function in antiviral and antitumor immunity depends on their proinflammatory cytokine IFN-*γ* or their direct killing of infected or transformed target cells such as virus-infected hepatocytes or hepatocellular carcinoma. They have both inhibitory and stimulatory receptors which act on their corresponding ligands on target cells [227]. NK cells inhibitory receptors include killer cell immunoglobulin-like receptors (KIRs) and CD94/NKG2 which recognize MHC class I molecules on target cells and inactivate the function of NK cells. The activating receptors include NKG2D, NCRs, and CD266 [220]. Thus, following acute viral infection, chemokines from hepatic resident cells recruit NK cells to inflamed liver and keep them in an activated state to control the infection. However, in chronic hepatitis C, studies have shown that NKG2 expression is increased on NK cells which may contribute to persistence of viral infection [221].

NK cells have also been suggested to be involved in preventing hepatic fibrosis, via killing-activated stellate cells which are key player in fibrosis due to its matrix deposition. Depletion of NK cells in experimental murine models enhances liver fibrosis [222]. 

NK cells may also be involved in hepatic tolerance. It has been reported that LPS-stimulated Kupffer cells secrete higher levels of the immunosuppressive cytokine IL-10, which in turn leads to inactivation of NK cell function [222]. NK cells may also indirectly maintain hepatic tolerance via dendritic cells which can induce tolerogenic regulatory T cells in the presence of NK cells [228]. 

### 3.9. NKT Cells

NKT cells are part of the innate immune system. They express both T-cell receptor and natural killer cell surface markers. They are a heterogeneous group which recognises lipid antigen presented by CD1d [229]. They are classified based on MHC class I like molecule, CD1d restriction as invariant NKT and noninvariant NKT cells. CD1d-dependent NKT cells are again classified into Type I and Type II NKT cells. Human NKT cells express TCR *αβ* or TCR *γδ* and a variety of NK cell receptors, which include CD161, CD69 and CD56 [230, 231]. 

Human intrahepatic NKT cells are defined as CD3^+^ CD56^+^ and consist of 10–15% of lymphocyte population but of that <1% is CD1d restricted invariant NKT. Intrahepatic NKT cells play an important role in defence towards hepatic infection or inflammation. Host antigen presenting cells present microbial glycolipid antigens to CD1d and NKT cells release Th1 (IFN-*γ*, TNF-*α*), Th2 (IL-4, IL-5, IL-10) or Th17 (IL-17, IL-22) cytokines which in turn activate other innate immune cells and adaptive T cells [232].

#### 3.9.1. NKT Cells and Hepatic Inflammation

NKT cells are enriched in liver and play a diverse role in acute liver injury, liver fibrosis and tolerance. It is due to different types of NKT cells and a variety of cytokines which they produce upon stimulation. In the acute injury setting, injection of *α*-GalCer, a specific ligand for invariant NKT will lead to acute hepatitis [233]. NKT cells also play a role in progressive fibrosis in nonalcoholic fatty liver disease both in human and murine models via activation of Hedgehog pathway [234]. NKT cells are implicated in hepatic tolerance. One elegant study suggested that IFN-*γ* secreted from NK cells upregulates CXCR3 ligands on hepatic sinusoid and subsequently recruits CXCR3 expressing regulatory T cells to control hepatic inflammation [235]. 

### 3.10. Innate Immune Cells Crosstalk Adaptive System in Hepatic Inflammation

Innate immune system provides signals to stimulate the adaptive immune system by proliferation and differentiation of antigen-specific T and B lymphocytes. Antigen peptide acts as a signal 1 which presents the antigen to [236] naive T cells via MHC-class II. Costimulatory molecules such as CD28, CD80, and CD86 are present on antigen presenting cells such as DCs to stimulate T lymphocytes thus acting as signal 2 to link the innate and adaptive immune response. Innate immune cells such as dendritic cells and macrophages produce polarizing cytokines in response to microbes that also promote the differentiation and growth of specific lymphocyte lineages. IL-12 stimulates naive T lymphocytes to develop into Th1 effector cells, IL-4 and IL-13 stimulate them into Th2 phenotype and IL-1, IL-6 and TGF-*β* into Th17 phenotype. Thus, polarizing cytokines in the microenvironment will shape the naive T cells into different T effectors lineages to counteract with different types of microbes (Figure 4).

### 3.11. Diagnostic and Therapeutic Clinical Application of Innate Immune Systems

Innate immune proteins and cells have been harnessed for many diagnostic and therapeutic applications in human diseases. Acute phase protein CRP, a mediator of inflammation and agent of innate immunity is now used as a key diagnostic marker of cardiovascular risk. Individuals with CRP levels <2 mg/L have significantly lower rate of coronary event. Thus, CRP levels are useful in evaluating the risk of myocardial infarction [237, 238]. Complement component levels are normally measured to assess the immune-mediated disorders and anaphylactic disorder such as hereditary angioedema. Tocilizumab, an anti-IL-6 therapy has been used in rheumatoid arthritis, cancer therapy, and cancer-related anorexia [239]. Cell therapy utilizing innate immune cells such as NK cells and DC is always an attractive option for clinical immunologists. Human NK cells immunotherapy is currently a promising tool as an adjuvant therapy in acute myeloid leukemia patients along with standard therapy [240, 241]. Furthermore, administration of myeloid DCs that have been pretreated with inactivated HIV enhances immune control of HIV in patients [242] and myeloid DCs pulsed with tumour antigen lysate (APF) induce tumour specific immune responses along with transarterial chemoembolization (TACE) in hepatocellular carcinoma patients [243]. 

Many GMP grade clinical trials are now underway for development of DC-based vaccine strategies in viral (HIV) and carcinoma (such as hepatocellular carcinoma) to elicit strong cytotoxic immune responses to overcome the immune regulation. However, vaccine strategies and cell therapies that aim to promote DC and NK cell responses during viral infection and antitumour therapy would have to be carefully monitored to prevent any deleterious consequences of immune activation. Gradual understanding of how DCs and NK cells are involved during viral infection at molecular level may provide new targets for vaccine design or even therapeutic modulation of disease with autologous cell therapy in future.

## Figures and Tables

**Table 1:** Toll-like receptors and their ligands, target microbes, and effector molecules are described.

TLRs	Ligands	Target microbes	Effector molecules
TLR1	Triacyl lipopeptides	Mycobacteria	Inflammatory cytokines
TLR2	Peptidoglycans, Lipoprotein; Zymosan	G+ bacteria Mycobacteria Yeast/other fungi	Inflammatory cytokines
TLR3	Viral double stranded RNA	Viruses	IFN*β*
TLR4	LPS	Gram-negative bacteria	IFN*β* Inflammatory cytokines
TLR5	Flagellin	Bacteria	Inflammatory cytokines
TLR6	Yeast zymosan Diacyl lipopeptides	Mycobacteria Yeasts and Fungi	Inflammatory cytokines
TLR7/8	Viral Single-stranded RNA	Viruses	IFN*α*
TLR9	Bacterial and viral CpG DNA	Bacteria/virus	IFN*α* Inflammatory cytokines
